# A systematic review and bioinformatic study on clinical, paraclinical, and genetic factors predisposing to stent restenosis following percutaneous coronary intervention

**DOI:** 10.1186/s12872-024-03955-3

**Published:** 2024-06-14

**Authors:** Farzad Shahsanaei, Abdullah Gharibzadeh, Soudabeh Behrooj, Shahin Abbaszadeh, Mahboobeh Nourmohammadi

**Affiliations:** Cardiovascular Research Center, Hormozgan University of Medical Sciences, Bandar Abbas, Iran

**Keywords:** Stent restenosis, ACS, PCI, Bioinformatic, Genes, MicroRNA

## Abstract

**Background:**

Stent restenosis is a relatively common phenomenon among patients with coronary heart disease undergoing percutaneous coronary intervention (PCI). It seems that a set of clinical, laboratory, and even genetic factors make people susceptible to such a phenomenon and in fact, this is multi-factorial. We aimed to first determine the underlying clinical and laboratory risk factors for the occurrence of stent re-stenosis after PCI based on a systematic review study, and after that, through a bioinformatics study, to evaluate the related genes and microRNAs with the occurrence of stent re-stenosis.

**Main text:**

In the first step, the manuscript databases including Medline, Web of Knowledge, Google Scholar, Scopus, and Cochrane were deeply searched by the two blinded investigators for all eligible studies based on the considered keywords to introduce clinical and laboratory determinants of stent re-stenosis. In the bioinformatic phase, and following a review of the literature to identify genes and microRNAs involved in restenosis, the interaction of each gene with other genes associated with stent re-stenosis was determined by GeneMANIA network analysis and Cytoscape software. Overall, 67 articles (including 40,789 patients) on clinical and biochemical predictors for stent restenosis and 25 articles on genetic determinants of this event were eligible for the final analysis. The predictors for this event were categorized into four subgroups patient-based parameters including traditional cardiovascular risk profiles, stent-based parameters including type and diametric characteristics of the stents used, coronary lesion-based parameters including several two target lesions and coronary involvement severity and laboratory-based parameters particularly related to activation of inflammatory processes. In the bioinformatic phase, we uncovered 42 genes that have been described to be involved in such a phenomenon considering a special position for genes encoding inflammatory cytokines. Also, 12 microRNAs have been pointed to be involved in targeting genes involved in stent re-stenosis.

**Conclusions:**

The incidence of stent re-stenosis will be the result of a complex interaction of clinical risk factors, laboratory factors mostly related to the activation of inflammatory processes, and a complex network of gene-to-gene interactions.

## Background

Ischemic coronary heart disease is the result of an imbalance between blood distribution and tissue demand in the myocardial muscle. Coronary artery lumen narrowing due to atherosclerosis is responsible for about 98% of ischemic heart disease cases [1]. It should be noted that coronary heart disease mainly affects the age group of 35 to 65 years, and in an important part of society, conflict may occur at the level of young people. In addition, coronary heart disease accounts for 64% of all cardiovascular deaths. According to the published statistics, during the last decade, we have seen an increase in the morbidity caused by ischemic cardiovascular diseases [2]. Of course, it should be kept in mind that with the development of medicinal methods as well as therapeutic interventions such as angioplasty and coronary stenting, the frequency of morbidity and mortality cases caused by these diseases has decreased significantly [3]. The first case of stent implantation was performed in 1986 and after that percutaneous coronary intervention or PCI was listed as one of the standard treatment strategies for these disorders [4]. Today, these methods as endovascular treatments aimed at restoring coronary blood flow have led to the lives of millions of people. However, observations have shown that within 6 months to one year after successful coronary stenting, there is a possibility of angina recurrence due to restenosis of the stent [5]. This issue has even been reported for drug-eluting stents [6]. After initial coronary stenting, the prevalence of restenosis is between 20 and 30% [7]. This stent restenosis occurs for various clinical and even genetic reasons and it seems that a set of pathophysiological processes such as inflammatory processes, proliferation, genomic and epigenetic factors play a role in it [8]. But interestingly, the role of each of these factors can be very different in different societies. In particular, the impact of genetic factors is completely dependent on the demographic characteristics of that society. Today, all kinds of genes, genetic polymorphisms, and microRNAs have been identified and introduced in the incidence of stent re-stenosis, which, along with clinical risk factors, double the risk of this complication. We aimed to first determine the underlying clinical and laboratory risk factors for the occurrence of stent re-stenosis after PCI based on a systematic review study, and after that, through a bioinformatics study, to evaluate the related genes and microRNAs with the occurrence of stent re-stenosis.

## Materials and methods

### Systematic review phase

**Fig. 1:**
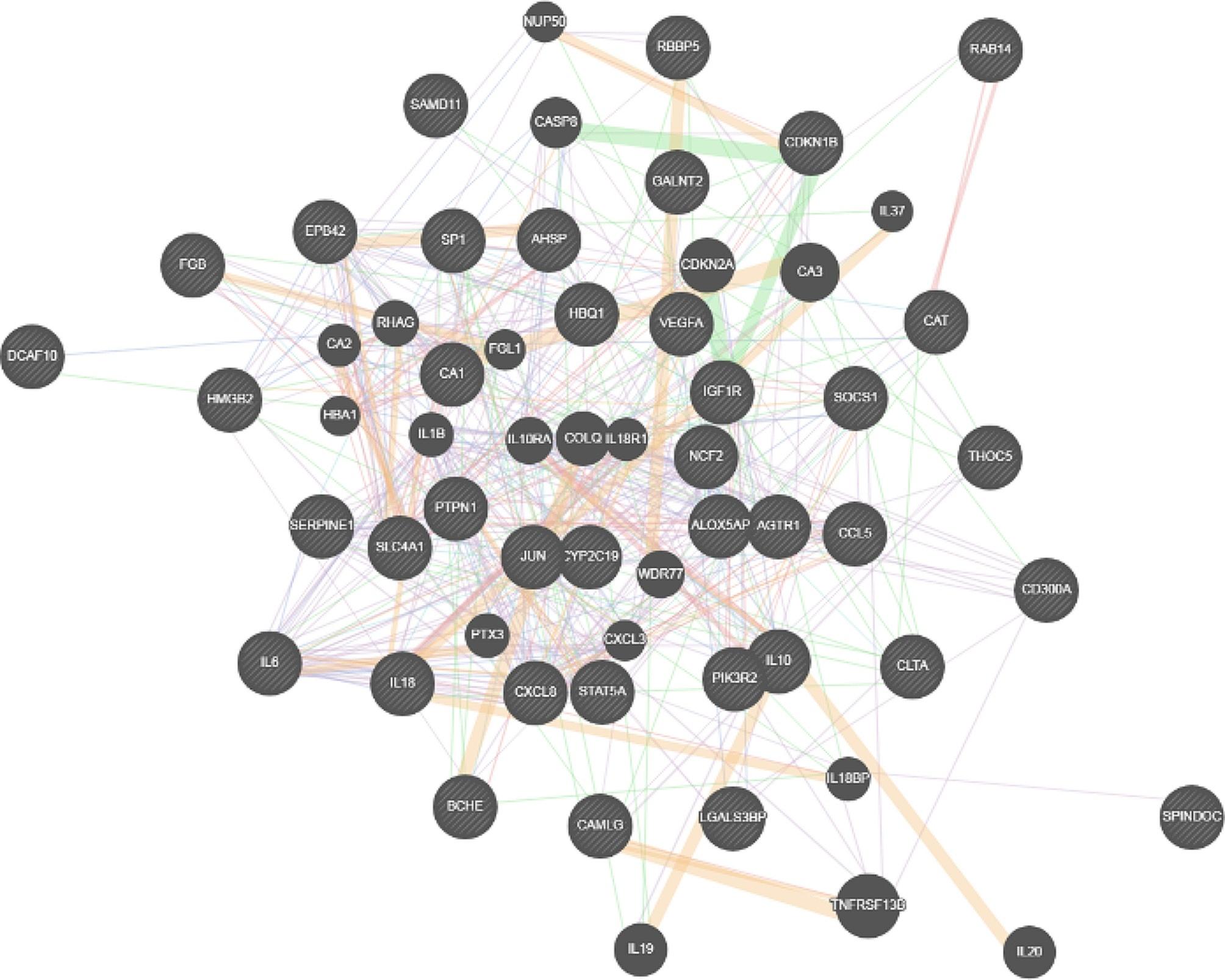
gene to gene interaction involving stent restenosis

The present systematic review and meta-analysis followed the guidelines for the Preferred Reporting Items for Systematic Review and Meta-Analysis (PRISMA). Firstly, two questions were suggested based on the author’s purposes “What are the main clinical determinants for stent re-stenosis?” and “What are the related and predictive genetic factors for re-stenosis?”. In the next step, the manuscript databases including Medline, Web of Knowledge, Google Scholar, Scopus, and Cochrane were deeply searched by the two blinded investigators for all eligible studies based on the considered keywords including “stent”, “stenosis”, “re-stenosis”, “predictor”, “risk factor”, “gene”, “genetics”, and “microRNA”. The inclusion criteria were considered to retrieve the studies: (1) the studies finally assessed clinical and/or genetic-based risk profiles related to stent re-stenosis, (2) due to the potential effects of other cardiac revascularization procedures such as coronary artery bypass grafting, those studies entering the patients who undergo such revascularization procedures or previous history of cardiac procedures were all excluded from analysis, (3) The studies were restricted to the English language, (4) the studies with unclear or irreproducible results were all excluded, (5) lack of access to the manuscripts full texts were also considered as the inclusion criteria unless the abstracts had enough data for our analysis, (6) case reports, case series, and review papers were all excluded. As shown in the flow diagram of the study selection (Fig. 1), 1536 articles were initially collected by database searching. After removing 13 articles due to evidence of duplication, 1523 records were primarily under-screened. Based on the titles and abstracts, 1426 records were excluded and the remaining 97 citations were assessed for further eligibility. Of those, 5 were also excluded due to the incompleteness of the data and contents. In the final, 67 articles on clinical and biochemical predictors for stent restenosis [10–76] (Table 1) and also 25 articles on genetic determinants of this event were eligible for the final analysis [77–101] (Table 2).

Data abstraction was independently performed by two un-blinded reviewers on structure collection forms without divergences in data collection. We resolved disagreements by consensus or by involving a third person. The study quality was evaluated based on the following criteria: (1) the systematic review and meta-analysis based on the questions primarily described and formulated; (2) inclusion and exclusion criteria predefined in the studies as eligibility criteria; (3) searching the literature performed on a systematic and comprehensive approach; (4) to minimize the bias, the full texts of the article were dually reviewed; (5) the quality of included studies were rated independently by the reviewers for appraising internal validity; (6) studies’ characteristics and findings were comprehensively listed; (7) the publication and risk of bias were listed; and (8) heterogeneity was also assessed. The risk of bias for each study was assessed using the criteria outlined in the Cochrane Handbook for Systematic Reviews of Interventions and also according to the QUADAS-2 tool. Any disagreement was resolved by discussion with the whole study team.

### Bioinformatic phase

The details of bioinformatic processing to assess the genes and gene-gene interactions are described by Sheikhvatan et al. previously [102]. Briefly, the interaction of each gene with other genes associated with stent re-stenosis was determined by GeneMANIA software (https://genemania.org/) indexing 2277 association networks containing more than 500 million interactions mapped to 163.599 genes in humans. The interactions were calculated based on FDR (False Discovery Rate) and coverage was classified under four categories (a) Shared protein domains, (b) Co-expression, (c) Co-localization, and (d) Genetic interactions. In this regard, a FDR ≤ 5% was considered to be significant. To design an integrated model of a gene interaction network, the Cytoscape software (version 3.6.1.0) was applied.

## Results

### Findings of a systematic review

To assess the main correlates of stent re-stenosis based on applied keywords, in total 67 studies finally assessed that published from different countries between 2004 and 2022 (Table 1). According to our risk of bias assessment, all 67 studies yielded good quality and none of the citations was determined to have a high risk of bias therefore the pooled results should be persuasive. In total, 40,789 patients were assessed and scheduled for primary stenting for coronary artery disease and followed up for 6 to 36 months after the procedure for assessing the occurrence of stent restenosis and its main determinants. The predictors for this event might be categorized as the four subgroups including (1) patient-based parameters including history of diabetes mellitus, hypertension, hyperlipidemia, smoking, history of tenting, chronic renal failure, history of non-alcoholic fatty liver disease, higher age, medical history of COPD, history of PCI, higher body mass index, low physical activity, Type D personality, anger, and some nutritional habits including lower folate intake, low fruit intake, low vegetable intake, and low vitamin C ingestion; (2) stent-based parameters including type of stent (BMS versus DES), lower stent diameter, longer stent, (3) coronary lesion-based parameters including two target lesions, Gensini score, TIMI score, coronary artery calcium score, coronary artery diffuse disease, peripheral vascular lesions, bifurcation lesion, CHA2DS2-VASc score, calcified plaque volumes, plaque burden, remodeling index, multiple stenting, stents in left anterior descending artery (LAD), and SYNTAX score; and (4) laboratory-based parameters including Higher HbA1c level, higher HsCRP level, raised ApoB, MCV and MCH values, higher neutrophil to lymphocyte ratio, higher apoA-I, higher Homocysteine, higher IgE level, increased lipoprotein-associated phospholipase A2 (Lp-PLA2) and IL-6 levels, higher monocyte count, raised creatinine, raised blood uric acid, lower high-density lipoprotein, higher S100A12 level, higher postoperative homocysteine level, higher VLDL-C, higher PDW, higher BMP-2 level, higher lymphocyte-to-Monocyte Ratio, lower serum albumin, higher white blood cell and neutrophil counts, higher lipoprotein A, higher serum IL-33 serum level, higher serum total bilirubin, higher serum Cystatin C, higher fibrinogen levels, higher serum sLOX-1 level, higher serum IL-6, lower serum IL-10, lower adiponectin levels, higher plasma heparin cofactor II activity, and insulin resistance (Table 1).

**Table 1:** Reviewing the studies on the predictors of stent restenosis

Author, year	No. population	Type of stent	Time after PCI (months)	Risk profile
Lin, 2022 [10]	797	DES	6	Higher HbA1c
Feng, 2022 [11]	235	DES	12	Diabetes mellitus, hypercholesteremia, SUA, HsCRP levels, two target lesions
Wang, 2022 [12]	472	BMS	12	Age, hypercholesteremia, raised ApoB, MCV & MCH values, Gensini score, diabetes mellitus
Csató, 2022 [13]	653	BMS, DES		neutrophil to lymphocyte ratio, type of stent, lower stent diameter, longer stents
Wang, 2022 [14]	604	DES	12	apoA-I, HbA1c
Wang, 2022 [15]	564	BMS, DES	12	CRP, HbA1c, lower QKI and COX-2
Zhou, 2022 [16]	215	BMS, DES	36	High RDW, lower stent diameter,
Guo, 2022 [17]	155	DES	24	High Homocysteine
Yi, 2022 [18]	1741	DES	12	Lower GFR, higher FBS, multivessel coronary disease, coronary artery diffuse disease, PCI operation time, emergency PCI, HbA1c
Liu, 2022 [19]	416	DES	12	Higher IgE and CML levels
Chen, 2022 [20]	452		12	Increased lipoprotein-associated phospholipase A2 (Lp-PLA2) and IL-6
Qiu, 2022 [21]	4392	DES	12	monocyte count
Luo, 2022 [22]	477	DES		remnant cholesterol, GS score, medical history of COPD, monocyte
Luo, 2022 [23]	429	DES	12	creatine, history of diabetes, smoking, multi-vessel lesions, peripheral vascular lesions, and blood uric acid
Zhang, 2022 [24]	284	DES	12	Lower AGR
Chen, 2022 [25]	257	DES	12	smoking history, higher fibrinogen
Zheng, 2022 [26]	194	DES		coronary artery calcium score
Li, 2022 [27]	341	DES	34	Lower LVEF, stent number
Hu, 2021 [28]	538		12	Anemia, diabetes mellitus, chronic kidney disease, multiple stenting, bifurcation lesion, calcification
Yoshimura, 2021 [29]	86	BMS	6	Hypertriglyceridemia, lower diastolic blood pressure, lower high-density lipoprotein
Gai, 2021 [30]	968	BMS, DES	12	platelet distribution width (PDW), total cholesterol, systolic blood pressure, low-density lipoprotein cholesterol, lesion vessels
Zhang, 2021 [31]	114	DES	9	stent length ≥ 35 mm
He, 2021 [32]	463	DES	12	prior PCI, hyperglycemia, stents in left anterior descending artery (LAD), stent type, absence of clopidogrel
Alexandrescu, 2021 [33]	235	DES	12	smoking, hypertension, diabetes mellitus, high CRP levels, CKD, TIMI score, stent type, low pressure for stent implantation, multi-stenting
Lee, 2021 [34]	1376	DES	12	severe chronic kidney disease
Sheng, 2021 [35]	847	DES	36	Higher Lp-PLA2
Gupta, 2021 [36]	550	DES		diabetes mellitus, deployment of stent in the left anterior descending (LAD) artery, periprocedural complication during percutaneous coronary intervention
Wang, 2020 [37]	209	DES	12	Hypertension, diabetes, number of coronary artery lesions ≥ 2, LDL-C ≥ 1.9 mmol/L, unstable angina, left anterior descending artery, diameter of stent ≥ 3 mm, length of stent > 20 mm
Wang, 2020 [38]	230	BMS, DES	12	Type D personality, low fruit intake, low vegetable intake
Liang, 2020 [39]	2443	DES	12	S100A12 level
Zhang, 2020 [40]	230	DES	12	Morisky score, anger, low physical activity, low folate intake, low Vitamin C ingestion
Tang, 2019 [41]	2338	BMS, DES	12	the number of stents
Sun, 2019 [42]	226	DES	34	Higher MCV, higher ALT, number of PCI vessels
Zhao, 2019 [43]	358	DES	12	stent diameter, Higher HbA1c
Cheng, 2019 [44]	1132	BMS, DES	12	hs-CRP levels, postoperative homocysteine levels, history of diabetes, coronary bifurcation lesions stent length
Baktashian, 2019 [45]	104	DES	12	Diabetes mellitus, stent type, serum hs-CRP, FBG, serum TG
Wang, 2018 [46]	368	DES	12	VLDL-C, UA, SYNTAX score, history of PCI
Hu, 2018 [47]	5232	BMS, DES	36	higher PDW
Wu, 2018 [48]	62	DES	12	post-procedural visfatin level, type 2 diabetes, reference vessel diameter, stent length, stent diameter
Wang, 2018 [49]	173	DES	24	Type D personality
Qin, 2017 [50]	1206	DES	24	Higher VLDL-C
Kurtul, 2018 [51]	358	DES	12	CHA2DS2-VASc score, total stent length, stent diameter, hs-CRP
Zheng, 2017 [52]	96	DES	12	BMP-2 level, diabetes, stent length, and stent diameter
Watanabe, 2017 [53]	131	DES	18	early generation DES, smaller stent, worse left ventricular contractility
Koiwaya, 2017 [54]	157	DES	12	smaller acute gain after initial ballooning, geographic mismatch between PCB position and initial ballooning, use of percutaneous transluminal coronary rotational atherectomy (PTCRA)
Yilmaz, 2017 [55]	1350	BMS	12	diabetes, hyperlipidemia, smoking, stent length, CHA2DS2-VASc score
Zhou, 2016 [56]	364	BMS, DES	12	epicardial adipose tissue volume
Tesche, 2016 [57]	74	DES	84	Calcified plaque volumes, plaque burden, remodeling index, lesion length
Murat, 2017 [58]	273	BMS	12	Lymphocyte-to-Monocyte Ratio, high-sensitivity C-reactive protein, stent diameter, stent length
Wihanda, 2015 [59]	289	BMS, DES	12	stent-type, stent length, bifurcation lesions, smoking, vascular diameter, hypertension, diabetes mellitus
Celik, 2016 [60]	341	BMS	12	Lower serum albumin, stent diameter, reason for stent implantation, body mass index
Bolca, 2015 [61]	404	DES	14	male sex, stent length, admission NLRs, white blood cells, and neutrophil counts
Park, 2015 [62]	595	DES	36	the reference vessel diameter, low-density lipoprotein cholesterol, total lesion length, Lp(a) ≥ 50 mg/dL
Yılmaz, 2015 [63]	675	BMS	12	platelet-to-lymphocyte ratio, serum C-reactive protein, smoking, diabetes mellitus, high-density lipoprotein, stent length
Zhao, 2015 [64]	529	DES	17	insulin resistance
Demyanets, 2014 [65]	387	BMS, DES	12	Serum IL-33 serum level
Shi, 2014 [66]	210	DES	12	Non-alcoholic fatty liver disease
Yildiz, 2014 [67]	131	DES	12	diabetes mellitus, stent length, preprocedural RDW, current smoking
Zhao, 2013 [68]	145	DES	11	serum total bilirubin, hs-CRP, Cystatin C
Aoyama, 2012 [69]	74	DES	12	chronic kidney disease
Lupi, 2012 [70]	267	BMS, DES	6	Higher fibrinogen levels
Munk, 2011 [71]	100	DES	18	flow-mediated vasodilation
Kuwano, 2011 [72]	1076	BMS, DES	12	Higher serum total bilirubin
Li, 2011 [73]	210	BMS, DES	12	Higher serum sLOX-1 levels
Zurakowski, 2009 [74]	73	BMS	12	serum CRP, IL-6, fasting glucose, lower IL-10
Kitta, 2008 [75]	148	BMS	12	low adiponectin levels
Takamori, 2004 [76]	166	DES	6	High plasma heparin cofactor II activity

### Findings of bioinformatic study

By reviewing 25 articles on genes involved we uncovered 42 genes that have been described to be involved in such a phenomenon. A complex network of genes, gene-related polymorphisms, and microRNAs were shown to be involved in increasing the likelihood of stent restenosis (Table 2). According to the literature, the up-regulation of some genes including JUN, SP1, RAB14, RBBP5, IGF1R, PTPN1, DCAF10, CLTA, CAT, STAT5A, CD300A, CA1, NCF2, HBQ1, AHSP, SLC4A1, EPB42, ADRβ2, CDKN1B, M2BP, CAMLG, GALNT2, C11orf84, THOC5, SAMD11, PIK3R2 SOCS1, VEGF, A1166C, HMGB2, BCHE, A1166C, CYP2C19, RANTES, ALOX5AP, SERPINE1, AGTR1, and FGB have been indicated by using different gene assessment techniques. Also, the predictive roles of the expression of some genes related to interleukin production (IL-18, IL-6, IL-10, and IL-8) have been highlighted. To determine the central role of the powerful genes related to stent restenosis, functional interactions and functional relationships between spike genes were evaluated by applying the Genemania network and Cytoscapre analytical software. As shown in Fig. 1, multiple pathways and gene-gene interactions seem to play a role in stent restenosis. In this context, many genes could interact with multi-pathway genes, but prominent gene interaction included co-expression (58.03%) followed by genetic interactions (13.28%). In this context, the main pathways activated in the background of this cluster based on FDR values were receptor signaling pathways via STAT with FDR value of 6.77e-8 (relevant genes of VEGFA, SOCS1, CCL5, IL10RA, IL18, STAT5A, and CD300A), cellular response to molecule of bacterial origin with FDR value of 6.77e-8 (relevant genes of HMGB2, IL1B, SERPINE1, CCL5, IL6, IL18, CXCL3, CXCL8) and response to lipopolysaccharide with FDR value of 6.77e-8 (relevant genes of HMGB2, IL1B, SERPINE1, CCL5, IL6, IL18, CXCL3, CXCL8). Along with gene polymorphisms and changing gene expression, some microRNAs were also assessed influencing genes and mRNA expressions that the studied microRNAs were shown in Table 1. In this regard, the special place of miR-139-5p, miR-324-5p, miR-513a-5p, miR-513a-5p, miR-525-5p, miR-548b-5p and miR-1253 (targeting the genes of JUN, SP1, RAB14, RBBP5, IGF1R, PTPN1 and DCAF10 respectively), miR-126-3p (targeting PIK3R2), and miR-30b-5p (targeting 62 genes related to vascular remodeling and fibrosis) has been shown.

**Table 2:** The genes, polymorphisms, and miRNAs related to stent restenosis

Author, year	Type of Study	miRNA	Targeted genes	Gene polymorphism
Song, 2022 [77]	Integrated microarray	hsa-miR-139-5p hsa-miR-324-5p hsa-miR-513a-5p hsa-miR-513a-5p hsa-miR-525-5p hsa-miR-548b-5p hsa-miR-1253	JUN SP1 RAB14 RBBP5 IGF1R PTPN1 DCAF10	
Chen, 2022 [78]	Integrated microarray		CLTA CAT STAT5A CD300A CA1 NCF2 HBQ1 AHSP SLC4A1 EPB42	
Abdelaziz, 2022 [79]	RT-PCR		ADRβ2 CDKN1B	rs1042713 rs36228499
Yang, 2022 [80]	RT-PCR		M2BP	
Liu, 2021 [81]	RT-PCR		CAMLG GALNT2 C11orf84 THOC5 SAMD11	rs12657663 rs2273970 rs643634 rs737976 rs9988179
Qiu, 2021 [82]	Bioinformatics analysis	hsa-miR-126-3p	PIK3R2	
Carretero, 2021 [83]	Integrated microarray	hsa-miR-30b-5p	62 genes related to vascular remodeling and fibrosis	
Maheronnaghsh, 2021 [84]	Integrated microarray	hsa-miR-152-3p		
Guan, 2018 [85]	Integrated microarray	hsa-miR − 1 hsa-miR − 21		
Zhou, 2017 [86]	RT-PCR		SOCS1 (gene promoter methylation)	
Bagyura, 2017 [87]	RT-PCR		VEGF	rs2010963 rs6999447
Zhu, 2017 [88]	DNA sequencing		A1166C	
He, 2017 [89]	RT-PCR		HMGB2	
Pleva, 2015 [90]	RT-PCR		BCHE	rs1803274
Li, 2015 [91]	RFLP assay		A1166C	
Nozari, 2015 [92]	RT-PCR		CYP2C19	CYP2C19*2
Liu, 2013 [93]	RT-PCR		IL-18	-137G/C
Gao, 2013 [94]	RFLP assay		IL-6	-572 C/G
Gao, 2011 [95]	RFLP assay		IL-10	-592 C/A
Vogiatzi, 2011 [96]	RFLP assay		IL-8	-251 A/T 781 C/T
Vogiatzi, 2009 [97]	RFLP assay		RANTES	-403G/A
Shah, 2008 [98]	RFLP assay		ALOX5AP	RS17222814 RS17216473 RS10507391
Katsaros, 2008 [99]	IHC		SERPINE1	
Wijpkema, 2006 [100]	RT-PCR		AGTR1	1166 A/C
Monraats, 2005 [101]	RT-PCR		FGB	-455G/A

## Discussion

Stent re-stenosis after primary stenting in patients with acute coronary syndrome is an uncommon but multifactorial phenomenon. This phenomenon is created and expanded as a result of the interaction of a set of clinical and laboratory factors as well as genetic predisposing factors. Obviously, due to the multifactorial nature of this incident, it will not be possible to accurately determine its prevalence. On the other hand, for the same reasons, it will not be possible to accurately estimate the occurrence of such an event. In this regard and based on a review of the literature, a wide set of background factors are involved in the occurrence of stent restenosis. Among the clinical factors, the presence of classic underlying risk factors of cardiovascular diseases has been completely predictable, in such a way that the risk of stent re-stenosis is higher in elderly patients, obese patients, hypertensive and diabetic patients, patients with hyperlipidemia, as well as patients with The history of chronic renal failure as well as the history of ischemic heart disease have been completely predictable. Also, among the laboratory risk factors, a special place can be given to inflammatory markers, because the occurrence of atherosclerosis is also the result of the interaction between underlying risk factors and inflammatory factors, and such a process can also be predicted in the case of stent re-stenosis. The basis of the occurrence of such a complication has a strong link with the activation of the inflammatory cascade. Additionally, the genomic polymorphisms have also provided the basis for the emergence and spread of stent restenosis. In this direction and during the last two decades, efforts have been made to identify the genetic factors related to the occurrence of this event using various genetic techniques, to identify types of gene polymorphisms, changes in the expression of various genes, and also to identify microRNAs related to it, which play an important role in the changes of the targeted genes involved. There is growing evidence of genetic contribution to vascular remodeling and ultimately coronary calcification and atherosclerosis through extracellular matrix changes and also MicroRNAs involvement in endothelial cell and vascular smooth muscle dysfunction in diabetic patients which makes it an interesting topic to evaluate in the context of in-stent restenosis.

## Conclusions

Therefore, it seems that the incidence of stent re-stenosis will be the result of a complex interaction of clinical risk factors, laboratory factors mostly related to the activation of inflammatory processes, and a complex network of gene-to-gene interactions, and therefore, it will not be possible to indicate on only one or a limited number of predisposing factors. However, special attention to some background factors can be considered. For example, identifying and tracking increased expression or discovering polymorphisms related to genes encoding various types of inflammatory interleukins can provide a way for early diagnosis and prevention of this complication. It is also obvious that controlling the traditional risk factors of cardiovascular diseases will be successful in preventing the occurrence of such a complication.

## Data Availability

All data generated or analyzed during this study are included in this published article, and further detailed ones are available from the corresponding author on reasonable request.

## Abbreviations

PCI: Percutaneous coronary intervention
PRISMA: Preferred Reporting Items for Systematic Review and Meta-Analysis
BMS: Bare-metal stent
DES: Drug-eluting stent
